# Supplementary material for: Communication training and the prescribing pattern of antibiotic prescription in primary health care
Source: PLoS One. 2020 May 19;15(5):e0233345. doi: 10.1371/journal.pone.0233345 (PMC7237035; doi:10.1371/journal.pone.0233345)
Supplement: Attachment — Submitted filename: Reply to Editor and Reviewers.docx [file pone.0233345.s003.docx]

**Reply to Editor and Reviewers, PLOS ONE, Submission PONE-D-19-15846**

**Communication training and the prescribing pattern of antibiotic prescription in primary health care**

Thank you very much for the careful and detailed reviews of our paper that were very helpful indeed for improving the initial submission. We incorporated (almost) all suggestions. Below you will find the specific responses to the editor’s and reviewers’ comments.

**Response to Editor Comments**

Additional Editor Comments (if provided):

The authors should be commended for their efforts to tackle a major public health problem, ATB over-prescription. The manuscript has been evaluated by two reviewers with expertise in the field of antibiotic use and methodology. As highlighted in their comments, the article needs in depth revision before it could be accepted for publication. Thus, while the article has the potential for publication, my decision is open rejection. If authors decide to re-submit their manuscript after in depth revision, it will be re-evaluated by the same reviewers.
**Response**: Thank you very much for the opportunity to re-submit our revised manuscript. The comments of the reviewer have helped us to improve the article a lot. We hope to have included the feedback to your satisfaction.

Major comments:

The introduction section is much too long and the way it is presented may discourage readers notably clinicians.

**Response**: Accordingly to the suggestions of the reviewer, we removed the development and the presentation of the hypotheses. Instead, some essential parts of the deleted material has been moved to the discussion section (as suggested by reviewer 2). As a result, the introduction is much shorter now.

Please justify the exclusion of pediatric patients which are at highest risk of ATB exposure.

**Response**: In this study we tested whether a communication training of primary care physicians affects their general antibiotic prescribing behavior. In Germany, the share of pediatric patients varies among primary care physicians depending where the practice is located. While in rural areas primary care physicians usually treat pediatric patients, in urban areas most pediatric patients are consulting pediatricians. To increase the comparability of the included cases and, thus, minimize a potential estimation bias of the communication training, we restrict the analysis to adult patients. We add this justification in the Materials and Methods Section.

Please support with references the strong assumption that “the average outcomes for the intervention and control group would have followed parallel trends over time before intervention”.

**Response**: This might be an issue of misunderstanding. We argue in the manuscript that the parallel trends assumption is implausible in our setting. Moreover, our data on average prescription rates for the intervention and control group, shown in Table 2, indicate this supposition. That is why we base our matching approach on past outcomes (average prescription rates). We provide references that this approach is a valid alternative identifying assumption allowing (under certain conditions) to interpret the Difference-in-Difference estimates as causal effects.

Please explain why the current design was not the one selected ahead (rather than the planned RCT) if it allows to identify “causal effect” and reproduces RCT “very well”.

**Response**: The reason was that we were not able to recruited sufficient participants to obtain the power needed to estimate a significant difference between the intervention and control group. In the new version of the manuscript, we explain why we formed the control group from observed data.

Please consider revising the wording used and stay more cautious regarding causality everywhere in the manuscript.

**Response**: You are very right. Although, we applied the combination of DID with matching and several robustness checks to minimize the risk of biased estimates, we cannot exclude that the results are biased. We therefore revised the manuscript accordingly to a reviewer’s suggestion by deleting the term “causal” and “demonstrate” when we are referencing to our estimated effects.

Please discuss the choice of a logistic modelling rather than a binomial or Poisson one.

**Response**: As the dependent variable, we considered the decision to prescribe an antibiotic for a specific URTI case. To account for the binary nature of the dependent variable we used a logistic regression model instead of count data models as negative binomial or Poisson models. Moreover, the logit model showed computational merits and, unlike the probit model, it did not suffer from any convergence failures. We add this justification in the Materials and Methods Section.

Please provide a rational for sample size.

**Response**: In the previous planned RCT and the corresponding published protocol, the minimum sample size has been calculated, yielding 31 per study arm. Since we have only be able to recruit 17 participants per study arm, we formed the control group from observational data including nearly all primary care physicians in private practices, working in a contract with statutory public health insurance. In the revised version of the manuscript, we describe in more detail why some physicians have been excluded from the study. An included flowchart with the number of physicians and the number of patients at each stage further helps the reader to follow our approach.

Please provide a flowchart.

**Response**: done!

**Response to comments of Reviewer #1**

Reviewer #1: Manuscript ID PONE-D-19-15846: Communication training and the prescribing pattern of antibiotic prescription in primary health care: A case-control study.

This manuscript reports the effect of a communication training intervention on the antibiotic prescription rate of primary care physicians (PCP) for the treatment of upper respiratory tract infections in adults.

The information reported in this manuscript is interesting and the statistical approach seem appropriate, on a subject which remains a major public health concern worldwide. However several points should be considered.

A) Major comments:

1) You state that your methodology (Difference-in-Difference estimation and matching approach) allows the demonstration of a causal relationship between the intervention and the outcomes. Such quasi-experimental designs seem to be a valuable option in numerous situations where the classic controlled randomized design is not feasible, and is clearly stronger than observational designs such as before-after studies. However, demonstrating a causal relationship remains particularly challenging, and statistical analysis comparing results from some RCTs to such DID finding similar results are probably not a sufficient proof (your reference 52). Even such RCTs often provide discordant conclusions, and sometimes only meta-analysis of well conducted RCTs allow providing the demonstration of a causal effect of an intervention. Moreover, to my knowledge, it is not recognized by international guidelines of level of evidence gradation, such as the GRADE system, that DID with matching is comparable to RCTs in term of level of evidence. Could you please provide clear support of this? If not, I strongly support removing this affirmation from all the manuscript (actually you used 8 times the term “causal” and 2 times “demonstrate”), including the abstract.

- **Response**: Thank you very much for your hint. You are right; we stated too loosely that we demonstrated a causal relationship. We applied the combination of DID with matching and several robustness checks to minimize the risk of biased estimates. However, of course, we cannot exclude that the results are biased with some accepted statistical error or even examine the risk that might be left. We therefore revised the manuscript accordingly to your suggestion by deleting the term “causal” and “demonstrate” when we are referencing to our estimated effects.

2) Your first hypothesis H1 is that your intervention could reduce inappropriate antibiotic prescriptions. However, all you analyses are focused to the reduction of overall antibiotic prescription rates, without detailing which prescriptions are appropriate or not. Moreover, you did not detail German guidelines on antibiotic treatments for URTIs in adults, to allow distinguishing appropriate and inappropriate antibiotic prescriptions. And finally in the discussion you stated that this hypothesis H1 is supported by your results.
I suggest to provide detailed data and analysis on which prescriptions were appropriate or not, in term of indication, class of molecule, duration of treatment, etc; or to change this hypothesis by “reducing overall prescription rate for URTIs”, which was the objective that you previously published in your protocol (your reference 50).

- **Response**: Thank you very much for this important comment. In the previous version of the manuscript, we have formulated our hypothesis too broadly. Unfortunately, our data set only provides the information about the diagnosis and whether an antibiotic has been prescribed. Therefore, we cannot distinguish between appropriate and inappropriate antibiotic prescriptions. We have implicitly argued that in the case of treating upper respiratory tract infections (URTIs) any antibiotic prescription is considered to be inappropriate. However, this is not true, as we have written in the manuscript “*For patients belonging to a higher-risk group (e.g., elderly patients) respective guidelines suggest the use of antibiotics in some cases*.” Moreover, in the revised version of our manuscript we describe in more detail the cases, where respective guidelines suggest the use of antibiotics (as suggested by reviewer 2). Therefore, we very welcome your suggestion to change our hypothesis to “*reducing overall prescription rate for URTIs*”. Since we have transferred and adapted the parts with the development of the hypotheses to the discussion, as suggested by the second reviewer, we have excluded the hypotheses from the text. However, we scrutiny adapted any misleading wording in regard to your suggestion that we are aiming to reduce the overall prescription rate for URTIs by means of the communication training.

3) Your initial aim was to conduct a randomized trial, but you stated that, due to lower inclusion rate than expected, and a lack of power, the analysis based on this trial was not statistically significant. Then, you reported that you “formed a control group from observational data” to apply a DID method and your matching. Your initial number of eligible PCP was 1554, but only 17 received the intervention. I don’t understand how many were allocated in the control group initially planned? None of them? The initial planned RCT had 3 arms, how much were in the third arm? Finally in the results section, you talk about 2189 eligible CPC? It is very difficult to follow it without any clear flow chart. If there was also 17 CPC in the initial control group, I don’t understand your matching, which was 1:1 if I well understood (It is not specifically mentioned) could increase your sample size? Finally, the total number of patients involved in the study is not clearly provided. A flow chart with the number of CPC and the number of patients at each stage would be very helpful. In the same way in the abstract, you said that 1554 PCP were invited to participate, but you neither report the number of PCP finally recruited, nor the number of patients.

- **Response**: Our study consists of two different analyses. In the first part, we conduct a pre-intervention analysis based on data from 2013 to 2015 of 2,189 PCPs. This is the number of enrolled physicians from 2013 to 2015 with non-missing data. In the second part of the study, we carried out the intervention analysis, which included data from 1,477 PCPs.

In the study protocol, the sample size has been computed to be 31 per study arm. In total, we have been able to recruited 34 physicians to participate in the study. Since the inclusion rate was much lower, both intervention groups have been consolidated. Splitting the group of 34 physicians resulted in 17 physicians that have been treated and 17 physicians that have assigned to the control group. However, due to the small number of recruited physicians even a comparison between the pooled intervention group and the control group had not resulted in statistical significant effects. That is the reason why we alternatively formed the control group from observational data consisting of 1,460 PCPs.

Since, we expect that the evolutions of the prescription rates are suspected to differ between the intervention and control group we apply a matching approach that is based on covariates and pre-intervention outcomes. The covariates are the same that have been used in the pre-intervention analysis. The additional conditioning on pre-intervention outcomes enables that all potential outcome trends are aligned between the intervention and control group.

In the matching approach, we do not apply a 1:1 matching. Instead, we extracted weights for each physician of the control group such that the control and the intervention group are balanced accordingly to pre-intervention out-comes and covariates. Physicians that are more similar to the trained physicians of the intervention group have higher weights. Therefore, we use all of the 1,460 PCPs of the control group; however, the specific PCPs are weighted accordingly to their entropy weights. The estimation procedure takes into account the weighting of the physicians and we did not just blow up the sample size.

We are aware about showing the results of both analyses (pre- and intervention) is much material for one manuscript. However, we prefer to show also the results of the pre-intervention analysis to increase the transparency. To ease the understanding we provide in the new version of the manuscript a flow chart with the number of PCP and the number of treated URT cases at each stage.

Other comments:

1) Could you define more precisely the study periods for each group? In the published protocol of the initial RCT, you planned to only include months from April to June, from 2013 to 2016. Was it the case in this study? If it is, could explain this choice? It is well known that most of the URTI diagnoses and prescriptions occur in the winter period.

- **Response**: Since in the first quarter of 2016 the training has been conducted, the periods initially planned to include in the study has been restricted to April to June. We could increase the sample length until the end of 2016. However, the first period of 2016 is still excluded from the study due to the training period.

2) Your main analysis report a significant reduction of antibiotic prescriptions, without detailing for which pathology. Do you have some precision about it? One concern when reducing antibiotic prescriptions is to increase the risk of treatment failure/complications. Do you have any data about hospitalization rate before and after the intervention, or the number of readmission?

- **Response**: No, unfortunately the data do not include any further information, e.g. about hospitalization. We only know the target-diagnoses of acute bronchitis, sinusitis and pharyngitis of the specific patient treated.

3) Another concern of such intervention is the long-term benefice. If I well understood, you assessed the effect of the intervention only few months after. Could you justify this choice? An important cluster randomized trials on antimicrobial stewardship showed that such intervention could have a transient effect (doi: 10.1001/jama.2014.14042), and without any continued feedback to clinician, the durability of the intervention could be jeopardized. Could you discuss it in the limits? Did you plan to provide feedback, which is an important component of antimicrobial stewardship?

- **Response**: This is a very good idea and of course an important concern for the limitations that we have now included in the new version of the manuscript. Thank you very much! Unfortunately, our choice is based on our data set. We only have data until the end of 2016. For future research, this is an interesting question.

4) Could you detail why among 1554 invited PCP, only 17 received the intervention? This is less than 1.5%, and could represent a substantial risk of selection bias, since the PCP who accepted to participate may be particularly aware about the need to reduce antibiotic use, while the remaining PCP, which constituted your control observational data, may be less interested in this. Could you discuss it in the limits? Again, this should lead you to be very careful in using the “causality” term, because this characteristic cannot be recorded in the baseline variables, and thus is very difficult, if not impossible, to account for in the matching analysis.

- **Response**: The risk of selection bias is exactly the reason why we applied the matching approach that is based on pre-intervention outcomes. After weighting the observations of the control group by the physician specific entropy weights, the outcome trends between the intervention and control group are similar as shown in Table 2. This means that physicians that have a similar prescribing behavior over time before the intervention have higher weights. Of course, we cannot guarantee that the applied matching takes into account the distinct awareness between the PCPs who accepted to participate and the ones who do not accepted. Therefore, we deleted the term “causal” and “demonstrate” when we are referencing to our estimated effects and add this issue to the limitations.

5) Could you explain why you choose only 3 CIM10 codes for URTIs, is there no other relevant code?

- **Response**: We concentrate the analysis to these diagnoses, since in the primary care setting for these cases an antibiotic is often prescribed, although only in some cases the use of antibiotics is suggested by respective guidelines within these diagnoses.

6) Your main analysis report a significant reduction of 6.5% of antibiotic prescriptions compared to the control group. Due to the major burden of antibiotic resistance,; even this small reduction may have an impact, but could you discuss, from an economic point of view, why you believe that this intervention may be more cost effective than another to reduce antibiotic use in the community?

- **Response**: We have trained primary care physicians to improve their communication skills, since literature suggests, that antibiotic prescriptions can be associated with a communication problem. Our results underline this hypothesis. Other studies have implemented different interventions like providing information material about antibiotic use for the patients or integrated a warning button in the physicians’ software. To assess the cost effectiveness of these interventions one needed to compare the effects and the underlying costs that are hardly available. However, we believe our intervention is cost effective because of two reasons. First, our intervention consists of a short (2 x 2.25h) communication training that can be easily integrated in advanced clinical education, as it is already part of under- and postgraduate training in the federal state of Schleswig-Holstein. Second, we believe that the effects of the communication training are not limited to the antibiotic prescriptions behavior. In future research, we plan to analyze the training effect on other behavioral aspects of the physicians, for instance withdrawals of proton-pump inhibitors prescriptions.

**Response to comments of Reviewer #2**

Reviewer #2: This study by Strumman et al. evaluates the effect of a communication training intervention on antibiotic prescribing for URTIs in German primary care settings. The authors used a difference-in-differences approach to evaluate prescribing between the intervention and control groups using both matched and unmatched approaches. The authors found that communications training resulted in a significant decrease in antibiotic prescribing for URTIs.
The study is well done and the methodology is robust. The findings align with previous studies of communications training and demonstrate the value of this intervention. At times, the complexity in the manuscript hinders the interpretability and impact of the study. Below are my comments, including some suggestions to help make the manuscript more interpretable.

Comments:

Introduction
• Consider if it is necessary to present all four hypotheses. It may suffice to state the main hypothesis and then note that that the study will also er. Having four hypotheses adds potentially unnecessary complexity to the manuscript.

**Response**: Thank you for this idea. We excluded the derivation of the moderation hypotheses and put some of these parts in the discussion.

• The arguments for the age and sex hypotheses also seem a little stretched and at times like you are pulling it out of thin air. For example, “women are less patient” is a ridiculous statement and doesn’t relate to antibiotic prescribing. However, the fact that reproductive-age women have more contact with the healthcare system than their similarly-aged male counterparts does probably relate gender differences in antibiotic prescribing. Also, early career for younger adults also likely has nothing to do with antibiotic prescribing. Age differences would more likely be related to increasing prevalence of co-morbid conditions with increasing age or a generational effect of changing culture around antibiotic use over time .

**Response**: In the new version of the manuscript, we deleted these speculative parts of the argumentation.

• Framing your paper with one objective would be more straightforward, allow you to remove the stretched relationships, and consolidate that information that would be more friendly to the reader.

**Response**: You are right. The new version of the manuscript now has a stronger focus on our main objective, i.e. the effect of the communication training on the antibiotic prescription behavior.

• I think moving most of the information on age and sex differences to the discussion may make this part of the discussion more robust and allow for a more streamlined and clear introduction. However, try not to stretch so much on the age and sex differences.

**Response**: We added some parts about age and sex differences from the introduction to the discussion. However, most of the material has been deleted due to reviewer comments.

• It could be helpful to provide a concise summary of guideline recommendations for antibiotic treatment (or non-treatment) for these conditions in Germany. In other countries, guidelines recommend antibiotic treatment for these conditions

**Response**: We add a concise summary of guideline recommendations in the Material and Methods section.

• Please double-check that reference 8 is the correct primary source for the information provided.
**Response**: We substituted the reference with a more appropriate one, i.e. Levy SB, Marshall B: Antibacterial resistance worldwide: causes, challenges and responses. Nat Med 2004, 10(12):S122-S129.

Methods
• It would be helpful to describe the ASHIP datasource more for readers who are unfamiliar. Who does it include? For example, what % of the population, what % of clinicians?

**Response**: We added two sentences to describe the ASHIP and the population/physician coverage.

• Did the intervention group know their data was being tracked? I am assuming the control group did not because it was observational data. Therefore, there could be a concern that behavior changed in the intervention group due to the Hawthorne effect rather than solely due to the intervention.

**Response**: Yes, of course you are right. We cannot exclude a Hawthorne effect. However, since we considered the data of the physicians up to one year after the training, it is less likely that this effect is responsible for persistent behavior changes. In the new version of the manuscript, we discuss this problem in the limitations.

• Is there a reason you did not include J03 tonsillitis in this study? Or J00 (common cold)?

**Response**: We concentrate the analysis to these diagnoses, since in the primary care setting for these cases an antibiotic is often prescribed, although only in some cases the use of antibiotics is suggested by respective guidelines within these diagnoses.

• Why did you use the number of URTI patients per year as a proxy for clinician workload instead of all patients per year or the total number of visits per year?

**Response**: This is due to data limitations. The data set provided consists only of URTI patients and do just not include the total number of visits. That is why we approximated that latter by the former assuming a proportional relationship between both numbers.

• How did you identify diagnosis certainty?

**Response**: We used the information provided by the billing data whether a definite diagnosis has been established or the diagnosis is coded by the physician as ‘suspected’. We reedit the description of the variable to increase the clarity.

• Please state software used for analyses.

**Response**: We add in the method section that the statistical analyses has been performed with STATA 15 (StataCorp LLC, College Station, TX, USA).

• Please briefly explain what the insurance categories mean for readers who are not familiar with German health insurance structure.

**Response**: In the measurements subsection of the methods section we added two further sentence to briefly describe the German health insurance structure.

• I am not sure that all the variables included as control variables are true confounders with relationships to both the exposure (communications training) and the outcome (antibiotic prescription). For example, is insurance status related to both the exposure and the outcome? For variables not related to both the exposure and outcome, it may be useful to explain why they were included in the model (for example, if they improved model fit).
**Response**: The insurance status, for instance, helps us to identify retired persons or coinsured family members. Both groups might be in different stages of their life (that cannot be fully depicted by age) with different expectations about an antibiotic prescription. Therefore, we consider this variable as an important confounder. In the revised version of the manuscript, this explanation is added. Moreover, the selection of potential determinants serving as control variables was based on related previous literature and on the pre-intervention analysis. All considered variables are found to improve significantly the model fit.

Results
• Table 2, with your population characteristics, should be the first table presented in the manuscript. All terms included need to be defined. For each characteristic, include all sub-levels. It could also be beneficial to move the current table 1 with regression coefficients to the supplemental material as this level of detail may be of interest only to a small portion of readers.

**Response**: Thank you very much for your hint to move the current table 1 to the appendix. However, from our point of view the multilevel modelling approach is an important part of the estimation strategy. That is why we (still) prefer to include the pre-intervention analysis in the manuscript due to increased transparency. Table 2 provides differences between the intervention and control group of the variables used for the matching. We have not included all sub-levels of the displayed characteristics, since they have not been all used for the matching due to collinearity. For instance, the share of cases of the first quarter is in exact linear relation to the share of cases of the other quarters. Sample characteristics for the pre-intervention analysis are included in Table 1. That is why we are showing this table at first. To reduce the complexity of Table 1, we removed two columns and, thus, only show the results of two distinct models.

• Although difficult with the complexity of your model, is there a simpler way to present the results from your DiD multilevel model? I think table 4 may be better suited for the appendix.

**Response**: Similar to Table 1, we deleted columns from Table 4. Only the results of the direct effect models and two models with the gender age interaction variables are shown. Although we place much lower emphasis on the gender-age aspect we (still) prefer to include the multi-level DiD analysis in the manuscript, because of the increased transparency and to test for gender-age specific differences of the intervention effect.

• I would suggest using only presenting one significance level as multiple can be confusing.

**Response**: To enhance the clarity we delete the 10%-level of significance. However, we believe that differencing between the 5%- and 1%-level adds important additional information for at least parts of the readers.

• In general all of the table columns and rows need better labeling so that it is clear what data you are presenting. I had a lot of trouble following. Please list referent groups for each subcategory in the tables too. Especially in Table 2 in share of cases (in %), it is really hard to following those options and to know what is the referent for each category. Use sub-headers to delineate that more clearly.

**Response**: Thank you for this advice. We add in tables 1 and 2 further sub-sub-headers to better guide the reader.

• Instead of saying that “H2 cannot be confirmed”, just state what you found. It is much clearer in plain language. This is a really good example of where the writing is overly complex and loses the reader, so that they can’t understand the excellent study you did.
**Response**: We droped the hypotheses from the manuscript.

Discussion
• Sections on the pre-intervention analysis in the results and discussion could be shortened dramatically as the real meat of this study is the effect of the intervention.

**Response**: Since we place much lower emphasis on the gender-age aspect, the description of the results and the discussion of the pre-intervention analysis has been shortened. However, in our point of view, the discussion of the findings of the pre-intervention analysis provides valuable insights about the antibiotic prescribing behavior that is helpful to evaluate the communication training effect and its moderation.

• The discussion was very nice and helped contextualize the complex findings.

**Response**: Thank you very much. We hope that following your suggestions, the complexity has been reduced and the new version offers a better understanding for the reader.

Overall/Other
• Check grammar and use of hyphens and use of dashes throughout.

**Response**: done!

• Consider changing the title as this is not a case-control study.

**Response**: Thank you. We deleted the term “case-control study” from the title.

• Please include copy of training materials (if possible) in supplement.

**Response**: The manual of the MAAS-Global-D has served as training material. It is available for free download at our webpage as stated and linked at the end of the conclusion section.

• The term “treated” is confusing as I am not sure if it refers to the visit or antibiotic treatment. Please rephrase where this term is used.

**Response**: In respective cases, we replaced the word treatment by consultation and visit.
