# Supplementary material for: Communication training and the prescribing pattern of antibiotic prescription in primary health care
Source: PLoS One. 2020 May 19;15(5):e0233345. doi: 10.1371/journal.pone.0233345 (PMC7237035; doi:10.1371/journal.pone.0233345)
Supplement: Attachment — Submitted filename: Response to Reviewers.docx [file pone.0233345.s004.docx]

**Response to Reviewers, PLOS ONE, Submission PONE-D-19-35281**

**Communication training and the prescribing pattern of antibiotic prescription in primary health care**

Thank you very much for the review of our paper. Again, it was very helpful indeed for improving the previous version of the manuscript. We incorporated (almost) all suggestions. Below you will find the specific responses to the reviewer’s comments.

Formularbeginn

Formularende

Review Comments to the Author


**Reviewer #1**

This is a well-written and clear manuscript that discusses an important antibiotic stewardship intervention with potential implications for both public health and clinicians which would be a helpful addition to the literature. Despite the small intervention sample size, I do think it is worth publication, and so have some suggestions for the authors to improve this manuscript. Additionally, I have very little experience with entropy balancing and using observational data as a control and, therefore, would recommend the journal consider a statistical review to ensure the approaches used were appropriate as I am unable to provide a full evaluation.

Suggestions:
• Throughout the paper, the authors reference inappropriate antibiotic prescribing. However, with the diagnoses listed here, there are some cases where antibiotic prescribing is appropriate (at least based on the information provided), so it is too speculative to call all prescribing for these conditions inappropriate. The authors either need to present more data about what proportion of these diagnoses might warrant antibiotic therapy (I am not sure if this data is available in the literature) or be very cautious and explicit in their interpretations.

**Response**: Thank you very much for this important comment. Of course, you are right; there are some cases where antibiotic prescribing is appropriate in cases of the listed diagnoses. We have described these cases in the *Data source* subsection. Unfortunately, our data set do not provide additional information that would enable us to assess the appropriateness of an antibiotic prescription. To make this point more explicit in the revised version of our manuscript, we indicate the aim of the study as to reduce the overall prescription rate for URTIs by means of the communication training. The term “inappropriate prescription” has been deleted to avoid any misleading wording.

• Introduction: Overall, a clear and concise summary of the evidence. At times, statements summarizing the literature seem over-generalized or too broad. This is especially pronounced on pages 3-4, starting with the sentence that begins with “Most patients possess insufficient knowledge…”. I believe all that is needed to ameliorate this is softening the language, for example, consider changing “Additionally, physicians wrongly assume to “Additionally, physicians may wrongly assume….”.

**Response**: At several stages, we have softened the language by inserting words as “may”, “might” etc.

• Introduction: I found the wording in the last paragraph of the introduction to be slightly confusing. Consider rephrasing and more succinctly stating the aims of the study.

**Response**: In the revised version of the manuscript, the aim of the study is stated more compactly.

• Methods: How were antibiotic prescriptions linked to diagnoses? Are indications listed for the prescription or was it inferred based on the visit diagnoses? If the latter, were visits with diagnoses for conditions where antibiotics are appropriate excluded (e.g., UTI, pneumonia)?

**Response**: Thank you very much for this important hint that we had not addressed in the previous version of the manuscript. We now have added the following paragraph in the *Data source* subsection. “Since the antibiotic prescriptions have been inferred based on the visit diagnoses, we excluded cases with additional diagnoses. This includes the presence of diagnoses regarding puerperium/pregnancy (O00-O99), further (bacterial) infections (A00 to A37, A39 to A79, J15, J17, J18) or chronic diseases (I50, J44, J45, C00 to C75). If the diagnosis had been made several times or more than one diagnosis had been made from the three groups (J01, J02, J20), the corresponding cases were also excluded.”

• Methods: I saw in the response to previous reviewer comments that tried to address the certainty of diagnosis variable, but I am still confused by this. It might be helpful to include more information about how this is recorded in the claim/medical record.

**Response**: We included further information about the German coding policy for primary care physicians regarding the certainty of the diagnosis in the *Measurements* subsection.

• Methods: were there any collinearity issues in your model when using both age and insurance states as these reflect similar “stage of life” clusters?

**Response**: Yes, there is an issue regarding collinearity, especially for the variables *Patient aged 65+* and *Pensioners insured*. If we neglect the variable *Pensioners insured,* the estimated effects of *Patient aged 65+*, *Female patient aged 35-65* and *Female patient aged 65+* are slightly stronger. All other effects are not affected. However, in our point of view, this collinearity problem is not critical, since it does not affect our main results. Moreover, we decided to include the variable *Pensioners insured*, since 3% of the observations are indicated as *Pensioners insured*, but are below the age of 65 and 1.5% of the observations are not indicated as *Pensioners insured* that are aged above 65. To summarize, we think that *Pensioners insured* serves as an important control variable and the collinearity issues can be neglected.

• Tables: I found the tables header in table 1 and 4 difficult to follow. I think it would be clearer to put a more descriptive title than just the model number, or if that is not feasible, include in a footnote the model specifics. This information is in the methods, but the tables need to be interpretable on their own.

**Response**: We extended the header and notes of the tables.

• Discussion: I think there might be other reasons for a declining trend in antibiotic use besides awareness of AMR. Consider other secular changes such as visit and disease incidence, antibiotic stewardship practices, and immunizations.

**Response**: Thank you very much for this hint. In the revised version of the manuscript, we extended the discussion by mentioning antibiotic stewardship programs. Since we included the number of URTI patients, the visit and disease incidence might be controlled for and do not serve as an explanation here.

• Discussion, page 16: The points about emergency medicine visits are fair, but the authors should also consider that these visits may be more severe than scheduled primary care visits.

**Response**: We integrated this important point in the discussion.

• I found the discussion of why younger adults and women might receive higher antibiotics speculative and unconvincing. Consider revising.

**Response**: We agree; this part of the discussion is somewhat speculative. However, our aim of the study was to gain further insights about the antibiotic prescribing process. Our hypothesis was that patients’ expectations are a key factor driving inappropriate antibiotic prescription due to a larger underlying communication problem. As we argue by means of the findings of other studies, the expectations might vary with age and gender. The underlying mechanisms behind these factors are far beyond our research focus. Therefore, the discussion remains here speculative. However, using these structural differences in the antibiotic prescribing behavior - potentially reflecting differences in the patients’ expectations - allows us to gain insights about differences in the effects of the communication training in the form that the higher the communication problem the more effective is an communication improvement. This result seems to be trivial, but it obtains insights of the underlying mechanism that is mostly not considered by other related studies.

However, to reduce the extent of the speculation, we revised the respective paragraph accordingly.

• Limitations: regarding the Hawthorne effect, did intervention clinicians know their data was being tracked? Were they aware of how long their data would be tracked? These are important considerations for understanding the extent of possible bias due to the Hawthorne effect.

**Response**: Yes, the participants did know that their data would be tracked before and after the intervention. Therefore, of course, we cannot control for the Hawthorne effect. This is an important limitation of our study that we discuss in the limitation section.

**Reviewer #2**

Manuscript ID PONE-D-19-15846: Communication training and the prescribing pattern of antibiotic prescription in primary health care: A case-control study.

Reviewer response: no additional comment.
